# Health and study dropout: health aspects differentially predict attrition

**DOI:** 10.1186/s12874-022-01508-w

**Published:** 2022-01-30

**Authors:** Johannes Beller, Siegfried Geyer, Jelena Epping

**Affiliations:** Hannover Medical School, Medical Sociology, Carl-Neuberg-Straße 1, 30625 Hannover, Germany

**Keywords:** Health, Chronic disease, Dropout, Attrition, Longitudinal

## Abstract

**Background:**

Participant dropout poses significant problems in longitudinal survey studies. Although it is often assumed that a participant’s health predicts future study dropout, only a few studies have examined this topic, with conflicting findings. This study aims to contribute to the literature by clarifying the relationship between different aspects of health and study dropout.

**Methods:**

The 2008 baseline sample of the German Aging Survey was used to predict study dropout (*N* = 4442). Indicators of health included physical health using the number of chronic conditions, physical functioning using the SF-36 Physical Functioning subscale, cognitive functioning using the digit symbol substitution test, and depression using the CESD-15.

**Results:**

It was found that different aspects of health had differential associations with survey dropout: Worse physical functioning and in part worse cognitive functioning predicted increased dropout rates; contrarily, worse physical health predicted decreased dropout when controlling for other health aspects and covariates. Depression was not significantly related to study dropout.

**Conclusions:**

Therefore, participants with chronic conditions, but minimal physical and cognitive disability were most likely to participate in the future. These findings suggest that health has a complex relationship with survey dropout and must be accounted for in longitudinal studies. Neglecting this systematic attrition due to health problems bears the risk of severely under- or overestimating health-related effects and trends.

## Background

Longitudinal studies, when the same participants are observed at multiple points in time, are vital to numerous areas of science, including Medicine, Public Health, Public Opinion Research, Psychology, Sociology, Econometrics and Political Science [1]. Conducting such studies allows the analysis of change over time in phenomena of interest, which is argued to be the basis for causal inference as one of the ultimate goals of science [2]. However, how well participants can be retained over time largely determines whether these goals can be achieved [3]. Participation attrition/Survey Dropout may lead to biased inferences and thus false conclusions thereby threatening the validity of longitudinal studies [4].

Although participant’s health is often assumed to be one of the most important predictors of dropout, only relatively few studies have analysed this purported relationship, with mixed results [3, 5–20]. For example, Banks and colleagues [5] analysed the relationship between several indicators of health, sociodemographic variables and attrition among older adults. They found that only the sociodemographic variables and not health predicted future attrition. Similarly, Wadsworth also found in their study of a national birth cohort that the response rates in general did not differ meaningfully between persons with and without serious physical illnesses [18]. Meneses and colleagues [8] analysed predictors of attrition among rural cancer survivors. In their study, physical health did not predict participant dropout; only mental health was found to be related to study attrition. Another study analysed predictors of attrition in Australian women [10]. In contrast to both previous studies, they found that physical health as well as mental health was significantly related to study dropout. As a last example, Goldberg and colleagues analysed predictors of attrition in a French cohort [14]. Among all of the studied predictors, physical health indicators had the strongest associations with future dropout in their study.

Therefore, although some previous studies had analyzed the relationship between health and study dropout, they provided mixed findings. Whereas some studies suggest that health does not significantly relate to survey participation, others suggest that health aspects are among the strongest predictors of study dropout. Additionally, studies from Germany are missing. So, more research is needed before firm conclusions regarding the potential contribution of health to survey dropout can be drawn [3]. As of yet, it might be speculated that worse health outcomes tend to be associated with increased dropout rates. The current study aims to examine how multiple aspects of health relate to survey dropout. It is examined which aspects of health—chronic conditions, physical functioning, depression, cognitive functioning—predict participant dropout using a large population-based sample of German middle-aged and older adults. We ask: How do the different aspects of health relate to future study dropout?

## Methods

### Sample

The 2008 wave of the German Aging Survey data was used, as described in a previous study [21]. This is a cohort-sequential longitudinal, population-based study on Germans above the age of 40 years. It was provided to the main author by the Research Data Center of the German Center of Gerontology [22]. The interviews were conducted face-to-face usually in the respondent’s place of residence and in the German language. National probability sampling was used for the German Aging Survey. All participants of 2008 who gave written consent were re-contacted whether they would participate in further waves of the survey in 2011, 2014, and 2017. To increase survey participation, participants were regularly contacted via information brochures and greeting cards, which were sent to the addresses of all participants. A broad range of information on the life of older adults in Germany is collected in the German Aging Survey, including their health status. In every follow-up data about mortality was also collected, which was based on the civil registry office and on reports of relatives of the deceased. Numerous studies have used the German Aging Survey for empirical studies on the health of middle-aged and older adults in Germany [23–28]. In this study data from all baseline participants of 2008 was used, who agreed to fill out a drop-off questionnaire, which resulted in a sample size of *N* = 4442.

### Measures

Several indicators of health in the 2008 wave were used to distinguish different aspects of health: number of chronic conditions, physical functioning, depression, and cognitive functioning. The number of chronic conditions was assessed via self-report with a list of conditions in a drop-off questionnaire (including heart disease, circulatory disorders, joint problems, respiratory disease, stomach or intestinal disease, cancer, diabetes, gallbladder, liver or kidney disease, bladder disease, sleep disorders, visual impairment, hearing impairment). Physical functioning was measured with the subscale Physical Functioning of the German version of the Short Form 36 Health Survey [29]. Depression was assessed with the 15-item German short version of the Center for Epidemiological Studies Depression Scale [CESD Scale;, [30]]. Cognitive functioning was measured via the German version of the Digit Symbol Substitution Test derived from the Wechsler Intelligence Test [31]. Regarding marital status for married participants and for those who indicated that they lived in a long-term partnership this variable was coded as 1 and for for all other participants as 0. Education was classified according to four levels: A low educational level (coded as 1) corresponds to participants who did not complete any vocational qualification and only had up to a maximum of a graduation degree. An intermediate educational level (coded as 2) corresponds to participants with vocational qualifications or participants who had the necessary qualifications for university entrance. An upper-intermediate educational level (coded as 3) corresponds to participants with a finished upgrading training, as for example is the case in Germany for a master craftsman. Finally, a high educational level corresponds to participants with completed university studies. Additional covariates included age, income (as percentage of the population average divided by 100), network size (as the number of important persons with whom the participant has regular contact) and sex. Similar to a previous study, information on future participation of baseline participants formed the basis of our dependent variable [21]. It was coded as 0 (did not drop out / participated in at least one further wave) or 1 (dropped out / did not participate in at least one further wave). Dropout could occur for several reasons including death, inability to contact, inability to respond, and insufficient motivation.

### Data analysis

Spearman correlation and logistic regression analyses were used to examine the degree to which different aspects of health predict future participant dropout, similarly to a previous study [21]. Missing values in the 2008 wave were imputed, because participants with missing values might be more likely to also drop out in further survey waves, thus decreasing the potential for bias [32]. The MissForest algorithm was used to impute missing values. It uses nonparametric random forests which seem especially useful to imputation of mixed-type data as in the current study. The missForest algorithm is able to outperform other imputation techniques [33]. Missing values were minimal for most variables except cognitive functioning (dropout: 0%; age: 0%; sex: 0%; education: 0%; physical functioning: 0%; network size: 0%; chronic conditions: 2%; depression: 2%; income: 8%; cognitive functioning: 21%). To assess the robustness of our results several additional analyses were performed. Firstly, because some previous studies suggested different dropout reasons for men and women, we provide sex-stratified analyses [34]. Additionally, instead of analysing a dichotomous dropout variable, we analyse how an alternative outcome, the number of times the participants participated in further waves, is related to our health indicators. Furthermore, we analysed a non-imputed list-wise deleted dataset. Lastly, since at least one study has found differences between predictors of dropout and predictors of mortality, we also conducted our analyses for a sample in which participants who are known to become deceased are excluded [5]. Thereby in the last sensitivity analysis, predictors of general survey dropout, for reasons other than mortality, are investigated.

## Results

Descriptive statistics and Spearman inter-correlations are reported in Table 1. As can be seen, participants (49% female) were on average 61.80 years old (*SD* = 11.88). 40% of participants dropped out and did not participate in successive survey waves. Indicators of health substantially and significantly correlated with each other (0.19 < = | r | < = 0.51). Descriptive differences between those who dropped out and those who continued to participate are depicted in Table 4 in Appendix.

**Table 1:** Descriptive Statistics and Inter-correlations of Dropout, Health Aspects and Demographic Variables (*N* = 4442)

	M / %	SD	(1)	(2)	(3)	(4)	(5)	(6)	(7)	(8)	(9)	(10)	(11)
1. Dropout (dropped out)	40%	–	–										
2. Participation count	1.37	1.29	−0.90***	–									
3. Chronic conditions	2.26	1.82	−0.03	0.00	–								
4. Physical functioning	84.57	22.07	−0.06***	0.08***	−0.51***	–							
5. Depression	6.35	6.20	0.03	−0.04*	0.30***	−0.39***	–						
6. Cognitive functioning	42.69	13.45	−0.13***	0.17***	−0.32***	0.37***	−0.19***	–					
7. Age	61.80	11.88	0.11***	−0.13***	0.40***	−0.43***	0.09***	−0.53***	–				
8. Sex (female)	49%	–	−0.01	0.03	−0.03	− 0.06***	0.08***	0.16***	−0.11***	–			
9. Education	2.49	0.96	−0.19***	0.20***	−0.08***	0.19***	−0.12***	0.27***	−0.16***	−0.19***	–		
10. Income	1.12	0.85	−0.18***	0.19***	−0.12***	0.21***	−0.20***	0.27***	−0.10***	−0.06***	0.40***	–	
11. Partnership status (has Partner)	79%	–	−0.08***	0.09***	−0.08***	0.16***	−0.18***	0.13***	−0.14***	−0.14***	0.16***	0.16***	–
12. Network size	4.40	2.83	−0.17***	0.18***	0.04**	0.02	−0.05***	0.10***	−0.12***	0.04*	0.08***	0.12***	0.10***

* *p* < .05; ** *p* < .01; *** *p* < .001. Higher values in chronic conditions and depression represent worse health. Higher values in physical functioning and cognitive functioning represent better health

*M* Mean, *SD* Standard deviation

Logistic regression results are presented in Table 2. Better physical functioning predicted decreased odds of dropout. The number of chronic conditions also significantly predicted decreased odds of dropping out and depression and cognitive functioning were not significantly related to dropout. Additionally, being older, female sex, lower levels of education, a lower income, a lower network size, and not being in a partnership predicted increased odds of dropping out. Of the health-related variables, chronic conditions and physical functioning had the largest standardized coefficients. These results were replicated when the isolated contribution of the health aspects was tested in separate regression analyses, as visible in Table 3.

**Table 2:** Logistic Regression Results Predicting Study Dropout via Baseline Health and Demographic Variables (*N* = 4442)

	OR	95%-CI	OR_standardized_	z	*p*
Chronic conditions	0.90	[0.86; 0.94]	0.82	−5.13	< .001
Physical functioning	0.99	[0.99; 1.00]	0.85	−4.06	< .001
Depression	0.99	[0.98; 1.01]	0.96	−1.10	.272
Cognitive functioning	1.00	[0.99; 1.00]	0.95	−1.21	.225
Age	1.01	[1.00; 1.02]	1.14	3.17	.002
Sex (female)	0.84	[0.74; 0.96]	0.84	−2.49	.013
Educational level					
Low (Ref.)	1.00	–	–	–	–
Intermediate	0.70	[0.57; 0.88]	0.70	−3.15	.002
Upper-intermediate	0.42	[0.32; 0.56]	0.42	−6.11	< .001
High	0.41	[0.32; 0.54]	0.41	−6.64	< .001
Income	0.76	[0.69; 0.86]	0.79	−4.65	< .001
Partnership status	0.87	[0.74; 1.02]	0.87	−1.73	.084
Network size	0.90	[0.88; 0.93]	0.76	−8.54	< .001

*OR* Unstandardized Odds Ratio, *95%-CI* 95% Confidence Interval of OR, *OR*_*standardized*_ Standardized (z-scaled) OR, *z* z-Value, *p* = *p*-value

**Table 3:** Logistic Regression Results Predicting Study Dropout via Baseline Health and Demographic Variables with Separate Analyses per Health Aspect (*N* = 4442)

	OR	95%-CI	z	*p*
Chronic conditions	0.92	[0.89; 0.96]	−4.07	< .001
Physical functioning				
Depression				
Cognitive functioning				
Age	1.01	[1.01; 1.02]	4.93	< .001
Sex (female)	0.84	[0.74; 0.96]	−2.57	.010
Educational level				
Low (Ref.)	1.00	–	–	–
Intermediate	0.68	[0.55; 0.84]	−3.56	< .001
Upper-intermediate	0.40	[0.30; 0.52]	−6.62	< .001
High	0.38	[0.30; 0.49]	−7.34	< .001
Income	0.75	[0.67; 0.84]	−4.99	< .001
Partnership status	0.85	[0.73; 0.99]	−2.03	.043
Network size	0.90	[0.88; 0.92]	−8.66	< .001
	OR	95%-CI	z	*p*
Chronic conditions				
Physical functioning	0.99	[0.99; 1.00]	−2.33	.020
Depression				
Cognitive functioning				
Age	1.01	[1.00; 1.01]	2.69	.007
Sex (female)	0.84	[0.74; 0.95]	−2.65	.008
Educational level				
Low (Ref.)	1.00	–	–	–
Intermediate	0.70	[0.57; 0.88]	−3.16	.002
Upper-intermediate	0.41	[0.31; 0.54]	−6.32	< .001
High	0.40	[0.31; 0.52]	−6.91	< .001
Income	0.77	[0.69; 0.86]	−4.55	< .001
Partnership status	0.87	[0.74; 1.02]	−1.71	.088
Network size	0.90	[0.88; 0.92]	−8.98	< .001
	OR	95%-CI	z	*p*
Chronic conditions				
Physical functioning				
Depression	1.00	[0.99; 1.01]	−0.67	.504
Cognitive functioning				
Age	1.01	[1.00; 1.02]	3.67	< .001
Sex (female)	0.84	[0.74; 0.96]	−2.50	.013
Educational level				
Low (Ref.)	1.00	–	–	–
Intermediate	0.69	[0.56; 0.85]	−3.39	< .001
Upper-intermediate	0.40	[0.30; 0.53]	−6.56	< .001
High	0.39	[0.30; 0.50]	−7.22	< .001
Income	0.76	[0.68; 0.85]	−4.79	< .001
Partnership status	0.85	[0.73; 1.00]	−2.00	.045
Network size	0.90	[0.88; 0.92]	−9.02	< .001
	OR	95%-CI	z	*p*
Chronic conditions				
Physical functioning				
Depression				
Cognitive functioning	1.00	[0.99; 1.00]	−1.16	.247
Age	1.01	[1.00; 1.01]	2.79	.005
Sex (female)	0.86	[0.75; 0.98]	−2.28	.023
Educational level				
Low (Ref.)	1.00	–	–	–
Intermediate	0.70	[0.57; 0.87]	−3.18	.001
Upper-intermediate	0.41	[0.31; 0.54]	−6.33	< .001
High	0.40	[0.31; 0.52]	−6.86	< .001
Income	0.77	[0.69; 0.86]	−4.60	< .001
Partnership status	0.86	[0.74; 1.01]	−1.86	.062
Network size	0.90	[0.88; 0.92]	−8.98	< .001

*OR* Unstandardized Odds Ratio, *95%-CI* 95% Confidence Interval of OR, *z* z-Value, *p p*-value

Also, we conducted several additional robustness checks: First, we analyzed women and men separately (Table 5 in Appendix). Here we find that similar associations as in our main analyses, except cognitive functioning. Higher cognitive functioning predicted decreased dropout strongly in women, but also predicted a slight increase in dropout in men. Second, we analysed the participation count instead of a binary dropout variable (Table 6 in Appendix). The participation count refers to the number of future waves the participants participated in. Here we find that, similar to the main analysis, chronic conditions, physical functioning, and cognitive functioning predicted increased chances of dropout. Third, we used a non-imputed list-wise deleted dataset (Table 7 in Appendix). Again, similar results as in the main analysis were found. And fourth, we excluded participants who died during follow-up, thus testing whether cases of mortality significantly influenced our potential conclusions (Table 8 in Appendix). Here we find that, again, higher chronic conditions and higher physical functioning significantly predicted decreased chances of dropout, thus suggesting strong robustness of our results.

## Discussion

Some studies had analyzed health as a predictor of study dropout but provided mixed results. Contributing to the literature by further investigating the relationship between health and study dropout, we considered four different aspects of health and investigated how these different aspects of health simultaneously predicted future study dropout in a large population-based sample. We found that the different aspects of health predicted dropout in a contrasting manner: Better physical and, in women, cognitive functioning predicted decreased odds of dropout, whereas fewer chronic conditions, and thus a better health status in terms of less chronic conditions, predicted increased chances of study dropout. Thus, participants who have chronic conditions but are not negatively impacted by them are most likely to participate in future survey waves. Depression did not show any association with dropout as soon as other health aspects were added to the model.

Our results support those studies that reported an association between health and study dropout [e.g., 10]. So, similar to some other studies we also found that health was strongly associated with survey participation [14]. At the same time, the findings of the current study contribute to the literature by showing that different aspects of health, having chronic conditions and the impairment associated with them, might have differential associations with future survey participation. To our knowledge, this is one of the first studies to show that worse health might be associated with increased as well as decreased chances for future dropout, depending on the health aspect. This might also explain the mixed findings regarding health-related dropout reported in the literature, where, surprisingly, physical health was also sometimes found not to be related to survey participation. Global measures of health might sometimes not predict dropout because the potential dropout-increasing associations of some health outcomes and the dropout-decreasing associations of other health outcomes are conflated in this summary measure. In contrast to some previous studies, depression was also not found to predict future study dropout, after controlling for other aspects of health [e.g., 8]. In consideration of the current results, overall associations of health with study dropout might not have been consistently observed in the literature, because they only emerge strongly once the diverging associations of different sub-aspects of health are disentangled, as has been shown in this and some previous studies.

How can these differential associations be explained? The literature has identified multiple main factors that predict survey participation and dropout [35]. Among them, ability (being able to participate) and motivation (being willing to participate) have to be distinguished [36, 37]. Likely, limitations in physical and cognitive functioning impair ones’ ability to participate in surveys and are thus associated with systematic dropout. Having chronic conditions, on the other hand, was associated with decreased odds of dropout. Having chronic conditions might increase one’s need to talk about these issues and might thus increase one’s willingness to participate in studies that address these topics. The lack of an association of depression with dropout in this study might be explained by the fact that depression represents a prevalent co-morbidity of physical morbidity [38]. In line with this hypothesis, depression was associated with dropout in the univariate analysis, but the association disappeared once one controlled for other aspects of health and demographic background information. However, future studies should disentangle the observed associations between aspects of health and study dropout even further, for example by distinguishing different sub-constructs of depression and emotional states.

The interpretation of longitudinal studies should be considered in light of these results. In contrast to previous work, strong associations of health with dropout were found. For example, one increase in a standard deviation of chronic conditions was associated with 18% decreased odds of dropping out, and each increase in a standard deviation of physical functioning was associated with 15% reduced odds of dropout. Therefore, participants seem to selectively participate in the further survey waves depending on their health. Biases in longitudinal studies might thus result in accordance to the degree that health is related to the phenomena of interest. For example, in longitudinal studies participants could seem functionally healthier than they are, all the while seeming to suffer from more chronic conditions and multimorbidity, although these biases might not extend to mental health [e.g., [39–42]]. Thus, authors of substantive research should be attentive to the potential health-bias inherent to longitudinal research.

Research employing longitudinal methods needs to account for this potential bias. One often-suggested strategy is to impute missing data [43]. Importantly, for this strategy to be feasible, multiple indicators of health need to be included that are able to account for the differential associations of health aspects with dropout. At least, indicators of chronic disease status and functional health need to be considered. Including only one overall health variable cannot account for the observed differential health-dependent dropout. Another strategy to perform longitudinal analyses and avoid this bias is to use other data sources that do not suffer from selective health dropout, like claims or health insurance data, which however might also be susceptible to other biases [44–46].

The current study could be improved in multiple ways. First, different types of dropout were not differentiated in the study, which might be needed to validate the supposed mechanisms. Similarly, due to the correlational nature of the research, causation cannot be ascertained. Although the current study statistically controlled for a range of potential covariates, there is still a risk of residual confounding. As such, future studies are needed that include an even more diverse set of variables in their analyses. Secondly, although the study used a large population-based sample of middle-aged and older adults, the sample did not include young adults. The associations of different aspects of health with attrition might differ in younger adults from middle-aged and older adults and should thus be analyzed by future studies. We also only included baseline health variables. Future studies might include time-varying health variables in a more complicated research design to study how dynamics of health development predict future dropout. In a similar vein, although several different health aspects were considered there could still be residual confounding. Therefore, future studies are needed that include further variables as potential predictors of study dropout. Furthermore, future studies should study whether similar results can be obtained when different study designs are used, such as is the case in RCTs or with different survey topics [e.g., [35, 47, 48]]. Lastly, it seems unclear why cognitive functioning seemed to predict dropout differentially in men and women, which should be investigated by future studies.

## Conclusion

The current study provides further evidence on how different indicators of health predict survey dropout. Participants with chronic conditions, but minimal physical and cognitive disability are most likely to participate in follow-up studies. Health has thus a complex relationship with survey dropout and must be accounted for in longitudinal studies to provide accurate research results. Neglecting this systematic attrition due to health problems bears the risk of severely under- or overestimating health-related effects and trends.

## Data Availability

The datasets supporting the conclusions of this article are available in the repository of the German Centre of Gerontology, https://www.dza.de/en/research/fdz/german-ageing-survey/data. However, restrictions apply to the availability of these data, which were used under license for the current study, and so are not publicly available.

## Appendix: Robustness analyses

**Table 4:** Descriptive Differences between Participants who Dropped out and those who continued to Participate in the Study (*N* = 4442)

	Stratified by Dropout			
	Level	Participated	Dropped Out	*p*
N		2651	1791	
Chronic Conditions (M (SD))		2.28 (1.74)	2.24 (1.92)	.486
Physical Functioning (M (SD))		86.52 (19.52)	81.68 (25.11)	< .001
Depression (M (SD))		6.15 (5.88)	6.65 (6.64)	.008
Cognitive Functioning (M (SD))		43.96 (12.92)	40.82 (14.00)	< .001
Age (M (SD))		60.76 (11.26)	63.35 (12.58)	< .001
Sex (%)	Male	51.0	52.2	.434
	Female	49.0	47.8	
Education (%)	Low	7.4	14.1	< .001
	Intermediate	50.1	60.4	
	Upper-Intermediate	14.3	9.6	
	High	28.2	15.9	
Income (M (SD))		121.36 (92.99)	99.93 (68.87)	< .001
Partnership Status (%)	Not in a Partnership	18.0	24.5	< .001
	In a Partnership	82.0	75.5	
Network Size (M (SD))		4.78 (2.81)	3.83 (2.76)	< .001

**Table 5:** Logistic Regression Results Predicting Study Dropout via Baseline Health and Demographic Variables (*N* = 4442) in Men and Women

	**Men**				
	OR	95%-CI	OR_standardized_	z	*p*
Chronic conditions	0.90	[0.86; 0.96]	0.84	−3.29	.001
Physical functioning	0.99	[0.99; 1.00]	0.84	−3.27	.001
Depression	1.01	[0.99; 1.02]	1.03	0.62	.537
Cognitive functioning	1.01	[1.00; 1.02]	1.15	2.68	.007
Age	1.01	[1.00; 1.02]	1.16	2.79	.005
Educational level					
Low (Ref.)	1.00	–	–	–	–
Intermediate	0.67	[0.44; 1.02]	0.67	−1.86	.063
Upper-intermediate	0.42	[0.26; 0.68]	0.42	−3.57	< .001
High	0.37	[0.24; 0.59]	0.37	−4.23	< .001
Income	0.73	[0.63; 0.85]	0.73	−4.04	< .001
Partnership status	0.89	[0.69; 1.13]	0.89	−0.97	.333
Network Size	0.92	[0.89; 0.95]	0.92	−5.10	< .001
	**Women**				
	OR	95%-CI	OR_standardized_	z	*p*
Chronic conditions	0.89	[0.83; 0.94]	0.80	−3.94	< .001
Physical functioning	0.99	[0.99; 1.00]	0.88	−2.28	.023
Depression	0.98	[0.97; 1.00]	0.90	−1.98	.048
Cognitive functioning	0.98	[0.97; 0.99]	0.79	−4.19	< .001
Age	1.01	[1.00; 1.02]	1.11	1.71	.086
Educational level					
Low (Ref.)	1.00	–	–	–	–
Intermediate	0.76	[0.58; 0.98]	0.76	−2.09	.037
Upper-intermediate	0.41	[0.28; 0.61]	0.41	−4.54	< .001
High	0.45	[0.32; 0.65]	0.45	−4.40	< .001
Income	0.79	[0.67; 0.94]	0.79	−2.71	.007
Partnership status	0.87	[0.70; 1.08]	0.87	−1.25	.211
Network Size	0.89	[0.86; 0.92]	0.89	−6.74	< .001

*OR* Unstandardized Odds Ratio, *95%-CI* 95% Confidence Interval of OR, *OR*_*standardized*_ Standardized (z-scaled) *OR*, *z* z-Value, *p p*-value

**Table 6:** Ordinal Regression Results predicting Participation Count in Further Waves (*N* = 4442)

	OR	95%-CI	OR_standardized_	z	*p*
Chronic conditions	1.08	[1.05; 1.12]	1.16	4.35	< .001
Physical functioning	1.01	[1.01; 1.01]	1.20	4.98	< .001
Depression	1.01	[1.00; 1.01]	1.03	1.01	.313
Cognitive functioning	1.01	[1.00; 1.01]	1.09	2.38	.018
Age	0.99	[0.99; 1.00]	0.91	−2.55	.011
Sex (female)	1.23	[1.09; 1.38]	1.23	3.40	.001
Educational level	–	–	–	–	–
Low (Ref.)	1.00	–	–	–	–
Intermediate	1.53	[1.25; 1.87]	1.53	4.15	< .001
Upper-intermediate	2.34	[1.83; 2.99]	2.34	6.80	< .001
High	2.45	[1.94; 3.09]	2.45	7.56	< .001
Income	1.19	[1.10; 1.29]	1.16	4.19	< .001
Partnership status	1.19	[1.03; 1.37]	1.19	2.39	.017
Network size	1.09	[1.07; 1.12]	1.29	8.83	< .001

*OR* Unstandardized Odds Ratio, *95%-CI* 95% Confidence Interval of OR, *OR*_*standardized*_ Standardized (z-scaled) OR, *z* z-Value, *p p*-value. Participation was measured as the number of future waves in which the baseline population was participating (0 to 3). The participation count refers to the number of future waves the participants participated in

**Table 7:** Logistic Regression Results Predicting Study Dropout via Baseline Health and Demographic Variables in a Listwise Deleted Dataset (*N* = 3155)

	OR	95%-CI	OR_standardized_	z	*p*
Chronic conditions	0.90	[0.85; 0.94]	0.81	−4.35	< .001
Physical functioning	0.99	[0.99; 1.00]	0.88	−2.65	.008
Depression	0.99	[0.98; 1.01]	0.96	−0.93	.351
Cognitive functioning	1.00	[0.99; 1.00]	0.94	−1.27	.204
Age	1.01	[1.00; 1.02]	1.11	2.13	.033
Sex (female)	0.87	[0.74; 1.02]	0.87	−1.67	.096
Educational level	–	–	–	–	–
Low (Ref.)	1.00	–	–	–	–
Intermediate	0.68	[0.52; 0.88]	0.68	−2.87	.004
Upper-intermediate	0.40	[0.29; 0.56]	0.40	−5.29	< .001
High	0.40	[0.29; 0.55]	0.40	−5.67	< .001
Income	0.81	[0.71; 0.92]	0.84	−3.23	.001
Partnership status	0.82	[0.68; 0.99]	0.82	−2.02	.043
Network size	0.90	[0.88; 0.93]	0.74	−7.41	< .001

*OR* Unstandardized Odds Ratio, *95%-CI* 95% Confidence Interval of OR, *OR*_*standardized*_ Standardized (z-scaled) OR, *z* z-Value, *p p*-value

**Table 8:** Logistic Regression Results Predicting Study Dropout via Baseline Health and Demographic Variables Excluding Participants Who Died during Follow up (*N* = 3967)

	OR	95%-CI	OR_standardized_	z	*p*
Chronic conditions	0.90	[0.86; 0.94]	0.83	−4.69	< .001
Physical functioning	0.99	[0.99; 1.00]	0.87	−3.31	< .001
Depression	0.99	[0.98; 1.01]	0.97	−0.81	.416
Cognitive functioning	1.00	[0.99; 1.00]	0.96	−1.00	.319
Age	1.01	[1.00; 1.02]	1.15	3.35	< .001
Sex (female)	0.84	[0.73; 0.97]	0.84	−2.43	.015
Educational level	–	–	–	–	–
Low (Ref.)	1.00	–	–	–	–
Intermediate	0.67	[0.53; 0.85]	0.67	−3.31	< .001
Upper-intermediate	0.40	[0.30; 0.54]	0.40	−6.06	< .001
High	0.37	[0.28; 0.49]	0.37	−7.03	< .001
Income	0.76	[0.67; 0.86]	0.79	−4.51	< .001
Partnership status	0.88	[0.74; 1.04]	0.88	−1.53	.127
Network size	0.90	[0.88; 0.92]	0.74	−8.43	< .001

*OR* Unstandardized Odds Ratio, *95%-CI* 95% Confidence Interval of OR, *OR*_*standardized*_ Standardized (z-scaled) OR, *z* z-Value, *p p*-value

## Abbreviations

CESD: Center for Epidemiological Studies Depression Scale
OR: Odds Ratio
CI: Confidence Interval
